# Personalized Parenteral Nutrition in Critically Ill Patients Undergoing Continuous Renal Replacement Therapy: A Comprehensive Framework for Clinical Practice

**DOI:** 10.3390/jpm15110545

**Published:** 2025-11-09

**Authors:** Nicola Sinatra, Antonino Maniaci, Giuseppe Cuttone, Tarek Senussi Testa, Simona Tutino, Daniele Salvatore Paternò, Alessandro Girombelli, Giovanni Ippati, Giorgia Caputo, Massimiliano Sorbello, Luigi La Via

**Affiliations:** 1 Nephrology and Dialysis Unit, “Paolo Borsellino” Hospital, 91025 Marsala, Italy; sinatra.nicola@libero.it; 2 School of Medicine and Surgery, “Kore” University, 94100 Enna, Italy; antonino.maniaci@unikore.it (A.M.); massimiliano.sorbello@unikore.it (M.S.); 3 Anesthesia and Intensive Care, ‘Abele Ajello’ Hospital, ASP Trapani, 91026 Mazara del Vallo, Italy; giuseppe.cuttone@hotmail.it; 4 Department of Cardiac Anesthesia and Intensive Care, Cardiovascular Network, IRCCS Policlinico San Martino Hospital, 16121 Genova, Italy; t.senussi@hotmail.it; 5 Department of Anesthesia and Intensive Care, Hospital “Giovanni Paolo II” ASP Ragusa, 97100 Ragusa, Italy; simona.tutino.29.9.92@gmail.com (S.T.); paternomd@icloud.com (D.S.P.); 6 Department of Anesthesia and Critical Care, SS Annunziata Hospital, ASLCN 1, 12038 Savigliano, Italy; alessandro.girombelli91@gmail.com; 7 Department of Anesthesia and Intensive Care, “S.A. Abate” Hospital, 91016 Erice, Italy; ippatigiovanni@gmail.com; 8 Department of Anesthesia and Critical Care, San Luigi Gonzaga Hospital, 10043 Orbassano, Italy; giorgia.caputo@ymail.com; 9 Department of General Surgery and Medical Surgical Specialties, University of Catania, 95123 Catania, Italy

**Keywords:** parenteral nutrition, continuous renal replacement therapy, CRRT, critical illness, personalized nutrition, intensive care unit, acute kidney injury

## Abstract

Critically ill patients receiving continuous renal replacement therapy (CRRT) face distinct nutritional challenges requiring specialized parenteral nutrition (PN) strategies. This review synthesizes current evidence with clinical expertise to provide a comprehensive nutritional framework for this population. Key findings reveal that CRRT significantly impacts nutrient homeostasis through daily losses of amino acids (14–22 g), water-soluble vitamins, and trace elements via the extracorporeal circuit. Results from observational studies demonstrate that higher protein targets (1.8–2.5 g/kg/day) are necessary to achieve positive nitrogen balance, while energy prescriptions must subtract “hidden” calories from citrate anticoagulation (3–4 kcal/mmol) and propofol (1.1 kcal/mL). Clinical outcome data, though primarily observational, indicate that achieving nutritional adequacy correlates with reduced ICU stays (average reduction 2.1–3.4 days), shorter mechanical ventilation duration, and improved functional recovery. Evidence supports that early PN prescription when indicated, coupled with systematic consideration of therapy modality, extracorporeal losses, oral intake capacity, and mobilization status, optimizes nutritional support. We conclude that successful implementation requires: (1) dynamic adjustment based on CRRT parameters, (2) integration with enteral nutrition when feasible, (3) regular metabolic monitoring, (4) multidisciplinary collaboration, and (5) structured protocols. Future research using point-of-care analysis and AI-driven support systems is needed to establish evidence-based guidelines in this specialized population.

## 1. Introduction

Nutrition is a cornerstone of supportive therapy in critically ill patients, as inadequate provision of energy, protein, electrolytes, vitamins, and trace elements may exacerbate catabolism, impair immune function, delay recovery, and worsen outcomes [1,2]. In the intensive care unit (ICU), enteral nutrition is generally preferred when feasible, but there remain subsets of patients in whom gastrointestinal intolerance or contraindications preclude adequate enteral feeding, making parenteral nutrition (PN) an essential alternative [3,4]. Among these, a particularly challenging group comprises patients with acute kidney injury (AKI) undergoing continuous renal replacement therapy (CRRT). CRRT is commonly employed in hemodynamically unstable critically ill patients and is associated with significant perturbations of fluid balance, solute clearance, and metabolic substrate handling [5,6]. The extracorporeal circuit and continuous modality of CRRT introduce unique metabolic interactions—particularly losses of amino acids, water-soluble vitamins, electrolytes, and glucose into the effluent—that can lead to underestimation of true nutritional needs if not carefully accounted for [5,7]. Moreover, the use of citrate anticoagulation or glucose-free dialysate/replacement fluids may lead to “hidden” caloric loads or deficits, further complicating energy and substrate calculations [5,6]. Despite the biologic plausibility of these influences and the clinical urgency of optimizing nutrition in CRRT patients, evidence-based guidance is scarce and most recommendations remain reliant on extrapolations or expert consensus [2,7,8]. The consequent risk is that patients may receive underfeeding, protein deficits, or unrecognized micronutrient depletion, all of which may adversely affect muscle preservation, immune competence, healing, and ultimately morbidity and mortality [1,3]. In this narrative expert review, we aim to articulate a personalized, physiology-informed framework for parenteral nutrition in critically ill patients undergoing CRRT, integrating available evidence, pathophysiologic principles, and practical considerations to support tailored nutritional therapy in the complex critical care environment.

## 2. Methods

This expert narrative review was conducted according to the methodological standards outlined in the Scale for the Assessment of Narrative Review Articles (SANRA), a six-item tool designed to improve the quality and transparency of non-systematic reviews [9]. SANRA emphasizes justification of the review’s relevance, explicit statement of aims, transparent reporting of the literature search, appropriate referencing, coherent scientific reasoning, and presentation of relevant data [9]. These six criteria served as the foundation for structuring our methodology. The primary aim of this review is to critically examine the principles of parenteral nutrition in critically ill patients undergoing CRRT, with a focus on personalization of therapy. In line with SANRA’s emphasis on clarity of objectives [9], this review does not attempt a systematic synthesis but rather provides an interpretive, expert-based integration of current knowledge, highlighting physiological rationale, clinical evidence, and gaps for future investigation. Following SANRA’s recommendation for transparent reporting of the literature search [9], we developed a structured but flexible search strategy. Searches were conducted in PubMed/MEDLINE, Embase, Web of Science, and Google Scholar (last updated July 2025). Search terms included: “parenteral nutrition”, “total parenteral nutrition”, “critical illness”, “intensive care”, “continuous renal replacement therapy”, “CRRT”, “acute kidney injury”, “nutrient losses”, “substrate kinetics”, and “hidden calories”. Boolean operators and both MeSH and free-text terms were applied. Reference lists of selected studies were also screened (“snowballing”), as recommended for comprehensive narrative coverage. Articles were included if they discussed parenteral nutrition in the ICU, CRRT-related metabolic alterations, nutrient and caloric losses, or clinical outcomes of nutritional support in this population. Studies exclusively on pediatric patients or intermittent dialysis were excluded unless their results were directly transferable to CRRT. Two independent reviewers screened all abstracts and full texts for eligibility, resolving disagreements by consensus or arbitration. Studies spanning observational research, interventional trials, clinical guidelines, and expert consensus papers were considered, consistent with the narrative scope supported by SANRA [9]. In keeping with SANRA’s focus on scientific reasoning and presentation of relevant data [9], extracted information included study type, population characteristics, CRRT modality and parameters, nutritional prescriptions, documented nutrient losses, and reported clinical outcomes. Data were synthesized thematically rather than quantitatively pooled. Contradictory findings were highlighted to avoid selective referencing. The narrative synthesis was performed through expert interpretation, contextualizing evidence within clinical practice and identifying gaps in knowledge. As SANRA recognizes the inherent risks of subjectivity in narrative reviews, steps were taken to minimize bias [9]. We actively sought contrasting viewpoints, supported all claims with primary evidence where possible, and highlighted methodological limitations of included studies. The limitations of this review itself—particularly its non-systematic design—are discussed explicitly in the conclusion, in line with SANRA recommendations.

## 3. Pathophysiological Considerations

Beyond the mechanistic challenges already outlined, the clinical consequences of unadjusted parenteral nutrition in patients on CRRT demand careful attention. In the presence of ongoing extracorporeal clearance, even modest under- or over-provision of energy or amino acids may translate into cumulative deficits or harmful excesses over days. Some observational data suggest that cumulative protein shortfalls are associated with worse outcomes in the ICU, including prolonged mechanical ventilation, loss of lean body mass, impaired wound healing, and increased mortality. For example, a narrative review of nutrition support in ICU patients with renal dysfunction emphasized that energy and protein targets must account for RRT-related losses, yet highlighted that clinical trials in this subgroup remain scarce and heterogeneous [2]. Moreover, experimental and clinical studies have quantified measurable losses of amino acids and proteins into CRRT effluent, which—if unaccounted for—may nullify attempts to achieve nitrogen balance. In a recent prospective study of low-dose continuous venovenous hemofiltration, median total amino acid loss over 240 min was 0.95 g, with protein losses (for those with detectable effluent protein) of ~2.52 g, underscoring that even short durations of therapy incur nonnegligible substrate loss [10].

Furthermore, the extracorporeal removal of micronutrients and water-soluble vitamins adds another layer of risk. Robust evidence shows that CRRT eliminates not only small molecules but also vitamins (e.g., B1, B6, folate, vitamin C), selenium, copper, and carnitine, with variable sieving or adsorption depending on modality, filter age, and flow rates [8,11,12]. In a mini-review on micronutrient losses during RRT, Lumlertgul et al. documented that water-soluble vitamins and trace elements are particularly susceptible to removal, and that the clinical implications of these losses remain underexplored [11]. Oh et al. further quantified losses of both amino acids and micronutrients across RRT modalities, emphasizing the importance of modality-specific adjustments [12]. These combined metabolite losses—if not compensated—may aggravate deficiencies, contribute to oxidative stress, and impair immunologic resilience in a population already vulnerable from critical illness.

Taken together, these lines of evidence underscore the imperative of a dynamic, individualized nutritional strategy in CRRT patients: one that is responsive to measured (or at least estimated) effluent losses, adaptable to changes in CRRT prescription (flows, anticoagulation, modality), and continually re-evaluated as the patient’s metabolic demand evolves. In the sections that follow, we propose a physiology-driven framework for parenteral nutrition tailored to CRRT, emphasising practical algorithms, monitoring guidance, and key research gaps.

## 4. Principles of Parenteral Nutrition in CRRT

When administering parenteral nutrition (PN) in patients undergoing CRRT, the clinician must carefully balance competing risks: underfeeding aggravated by extracorporeal losses, and overfeeding compounded by hidden caloric inputs. In this section, we present a physiology-based roadmap for PN in CRRT, addressing energy, amino acids/protein, lipids, glucose, and electrolytes/micronutrients, always emphasizing the need for dynamic adjustment (Table 1). Table 1 summarizes the key nutritional parameters requiring adjustment in CRRT patients, along with recommended targets and monitoring strategies.

### 4.1. Energy Requirements and Substrate Utilization

Estimating energy needs in critically ill patients is complex even in the absence of CRRT; the extracorporeal circuit further complicates matters by introducing non-nutritional caloric loads and substrate removal. Sedative agents like propofol, or citrate used for anticoagulation, may supply significant “hidden” calories that must be accounted for in the total energy prescription. In general critical care nutrition discussion, non-nutritional calories have been shown to contribute 6–18% of total energy intake in the first week (from propofol or citrate) [13]. These hidden caloric contributions must be precisely quantified: propofol delivers 1.1 kcal/mL (as a 1% emulsion, equivalent to approximately 110 kcal per 100 mL), while citrate anticoagulation provides 3–4 kcal/mmol of citrate (typically supplying 200–500 kcal/day depending on CRRT prescription and citrate concentration). For a 70 kg patient receiving citrate CVVHDF at an effluent rate of 25–30 mL/kg/hour with regional citrate anticoagulation (4% trisodium citrate solution), this can represent 10–15% of total daily caloric requirements. Similarly, if replacement or dialysate solutions contain glucose (typically 0.1–0.2%), this adds approximately 2 kcal per liter of solution used. These seemingly modest contributions become significant during continuous therapy, potentially resulting in unintended overfeeding if not explicitly subtracted from the nutritional prescription.

Thus, the PN energy target in CRRT patients should begin conservatively (e.g., 20–25 kcal/kg/day adjusted for ideal or adjusted body weight) and be modified by subtracting or adjusting for known non-nutritional calories (from citrate metabolism, dextrose in CRRT fluids, propofol) [13]. Indirect calorimetry remains the gold standard for quantifying resting energy expenditure, and when available should guide adjustment. Over time, energy prescription should be adapted to metabolic phase (e.g., early vs later critical illness), ventilatory tolerance (CO_2_ production), glycemic control, and observed trends in nitrogen balance.

### 4.2. Amino Acids and Protein Targets

One of the most critical considerations in CRRT is compensating for amino acid losses across the extracorporeal circuit. In a study of CVVH using modern polysulfone membranes, Stapel et al. quantified amino acid loss at ~13.4 g/day (~11.2 g protein equivalent) [14]. Davies et al. observed that critically ill AKI patients on CRRT may lose nearly 15 g of amino acids in a 24-h period [15]. These losses are nontrivial when the background catabolic state is considered.

To offset this, PN protein dosing in CRRT should exceed conventional ICU protein targets. While robust trial data are lacking, expert opinion and observational data suggest aiming for 1.8 to 2.5 g protein/kg/day (based on ideal weight or lean mass) in hypercatabolic CRRT patients, with upward titration if losses are sizable or if nitrogen balance remains negative. Practically, a starting target of ~2.0 g/kg/day may be reasonable, with close monitoring of urea nitrogen generation, amino acid levels (if available), and clinical response.

Regarding specific amino acid composition, standard parenteral amino acid formulations may be suboptimal for CRRT patients. Specialized renal-specific amino acid solutions, which typically contain higher concentrations of essential amino acids (particularly branched-chain amino acids like leucine, isoleucine, and valine) and reduced amounts of non-essential amino acids, may be advantageous. Histidine, lysine, and threonine losses are particularly significant during CRRT, with sieving coefficients approaching 0.9–1.0 in some studies [14,15]. Glutamine supplementation (0.3–0.5 g/kg/day), though controversial in general critical care, may merit consideration in CRRT patients with prolonged therapy due to documented substantial losses, though monitoring for potential toxicity remains essential.

### 4.3. Lipids: Role and Tolerance

Lipids serve as essential calories and provide essential fatty acids, while sparing dextrose burden. In ICU PN practice, maximum lipid infusion rates are often limited to avoid fat overload; guidelines typically recommend staying below ~1.5 g lipid/kg/day (or ≤0.1–0.15 g/kg/h) depending on patient tolerance [16,17]. The ESPEN/clinical nutrition consensus also supports use of mixed emulsions (e.g., combining long-chain, medium-chain, and omega-3 lipids) to reduce oxidative stress and inflammatory sequelae [18].

In CRRT patients, lipid emulsions are not significantly cleared by the extracorporeal circuit, so standard dosing constraints apply. However, because critical illness is associated with oxidative stress, lipid peroxidation is a known concern—antioxidants like vitamin E may be coadministered, and use of more stable lipid formulations (e.g., MCT/olive oil blends) is favored [1,16]. Triglyceride levels must be monitored; if hypertriglyceridemia arises, lipid infusion rates should be reduced, and caloric deficits compensated via dextrose or amino acid adjustment.

Specific lipid formulations for CRRT patients should be selected based on their immunomodulatory and anti-inflammatory properties. Third-generation lipid emulsions containing a mixture of soybean oil (30–50%), MCT (30–50%), olive oil (15–30%), and fish oil (10–15%) are preferable to pure soybean oil-based solutions. Fish oil components (eicosapentaenoic acid and docosahexaenoic acid) at doses of 0.1–0.2 g/kg/day may reduce inflammatory mediators particularly relevant in the context of AKI. Structured lipids, where medium- and long-chain fatty acids are attached to the same glycerol backbone, offer potential metabolic advantages through more efficient clearance and reduced liver fat deposition—a consideration in prolonged CRRT where hepatic dysfunction may coexist.

### 4.4. Glucose Management and Insulin Needs

Glucose infusion in PN must be tailored carefully in CRRT patients, because glucose may be lost via the CRRT circuit or introduced by dialysate/replacement fluids. Hence, the nominal glucose infusion rate (GIR) should be adjusted downward by estimated circuit glucose flux. In critical care nutrition guidelines, a GIR of ≤5 mg/kg/min is often recommended to minimize hyperglycemia [18].

Frequent glucose monitoring (hourly to bi-hourly initially) is essential, with insulin infusion titrated to maintain target glycemia (e.g., 140–180 mg/dL or institutional standard). Changes in CRRT prescription (flow rates, modality, buffer composition) should prompt reassessment of net glucose balance and insulin dosing. If hypoglycemia or hyperglycemia persists, PN dextrose or insulin rates must be recalibrated.

### 4.5. Electrolytes, Micronutrients and Vitamins

Continuous removal of electrolytes and small solutes is an intrinsic aspect of CRRT, risking deficits in potassium, phosphate, magnesium, calcium, sodium, and others. In PN for CRRT patients, electrolyte supplementation must be proactive: laboratory monitoring should initially occur every 4–8 h and drive adjustments in the PN admixture or supplemental infusions.

Micronutrient and water-soluble vitamin losses are well documented during CRRT. In a study of micronutrient clearance, Lumlertgul et al. measured daily losses of vitamins, amino acids, trace elements (e.g., zinc, selenium, carnitine) and found that >30% of CRRT patients had plasma micronutrient levels below reference ranges over six days of therapy [19]. Standard supplementation doses for these patients typically include selenium 100 μg/day, zinc 10–15 mg/day, and water-soluble vitamins at 1–1.5× the recommended daily allowance (thiamine 100 mg/day, pyridoxine 100 mg/day, vitamin C 100 mg/day, folic acid 1 mg/day), yet these may be insufficient. Likewise, the same investigators demonstrated significant carnitine depletion and reductions in citrulline and glutamic acid in CRRT patients [19]. These data underscore the need to supplement vitamins and trace elements (especially water-soluble ones) at or above normal ICU PN levels, often 1.5–2× standard doses, while exercising care with possible toxicity.

Fat-soluble vitamin loss is less pronounced acutely but should not be ignored in prolonged PN. Given the potential role of antioxidants (e.g., vitamin C, selenium, zinc) in modulating oxidative injury and immune competence, tailored supplementation is justified in high-risk CRRT populations—but always within upper safety limits and considering renal excretion capacity.

### 4.6. Albumin

The role of serum albumin and prealbumin (transthyretin) measurements requires special consideration in CRRT patients. While traditionally used as nutritional markers, both proteins are significantly affected by extracorporeal therapy. Albumin (half-life ~20 days) may be lost across high-flux membranes at rates of 3–5 g/day, while prealbumin (half-life ~2–3 days) demonstrates sieving coefficients of 0.3–0.4 in contemporary CRRT systems. Consequently, measured levels reflect both nutritional status and circuit-related losses. When monitoring these parameters, clinicians should target albumin levels of 3.0–3.5 g/dL and prealbumin of 18–22 mg/dL, understanding that values below these ranges may not necessarily indicate nutritional inadequacy but rather CRRT-specific depletion. Notably, albumin administration (typically as 20% or 25% solution at doses of 20–40 g/day) may be indicated not primarily as nutritional support but rather for intravascular volume maintenance, oncotic pressure support, and as a carrier for medications and endogenous substances including calcium, magnesium, and certain drugs. The rate of change in these proteins over time, rather than absolute values, provides more meaningful nutritional assessment in CRRT patients.

## 5. Personalized Approaches

**Figure 1:**
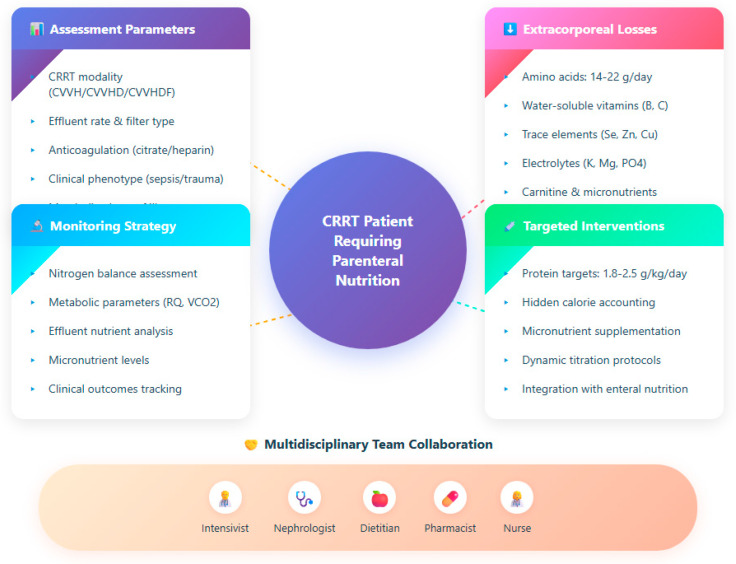
Integrated approach for nutritional management in continuous renal replacement therapy.

In patients undergoing CRRT, the paradigm of one-size-fits-all nutrition is inadequate: personalized strategies must continuously integrate CRRT modality, patient metabolic state (e.g., in sepsis or multi-organ failure), and dynamic monitoring feedback to tailor PN. In this section, we propose how to adapt parenteral nutrition according to CRRT settings, clinical phenotype, biomarker trends, and how to prevent overfeeding or underfeeding in this nuanced setting (Figure 1). Figure 1 illustrates the comprehensive framework for personalizing parenteral nutrition in CRRT patients, integrating assessment parameters, quantified losses, monitoring strategies, and multidisciplinary collaboration.

### 5.1. Tailoring Nutrition to CRRT Modality and Dose

Because CRRT modalities differ—continuous veno-venous hemofiltration (CVVH), hemodialysis (CVVHD), or hemodiafiltration (CVVHDF)—each with distinct convection/diffusion balance, the profile of nutrient removal changes accordingly. Higher effluent rates remove more small molecules (including amino acids, water-soluble vitamins), so patients on higher CRRT doses will require more aggressive compensatory nutrition. Vicka et al. reported that patients may lose 14 to 22 g of amino acids per day during CRRT, contributing substantially to negative nitrogen balance [20]. Therefore, when a clinician increases the effluent flow or switches to a more convective regime, the PN formula needs recalibration—particularly increasing amino acid supply proportionally, while carefully adjusting other substrates to avoid imbalance.

Furthermore, the filter membrane properties (e.g., pore size, adsorption capacity) and the mode of anticoagulation (e.g., citrate vs heparin) may influence solute clearance and “hidden” caloric loads. Thus, a tailored approach maps CRRT parameters to estimated nutrient losses and subtle energy transfers, and dynamically updates PN accordingly.

### 5.2. Adjustments for Sepsis, Trauma, and Multi-Organ Failure

In critical illnesses—like severe sepsis, trauma, or multi-organ failure—the metabolic stress response is magnified: there is greater proteolysis, lipolysis, insulin resistance, altered substrate use, and mitochondrial dysfunction. Personalization in such patients means adjusting not only for CRRT losses but also for heightened catabolic demand. The current literature on ICU nutrition emphasizes that personalizing nutrition—even in non-CRRT populations—enhances outcome potential (e.g., by phenotyping and endotyping patients) [21]. In septic patients, early permissive underfeeding (e.g., 70% of energy targets) has been advocated in some settings to reduce metabolic stress, yet in CRRT patients such underfeeding might exacerbate deficits unless protein is preserved [22]. In trauma, both early and higher protein delivery may favor healing, so one might adopt a strategy of moderate energy but elevated protein. In multi-organ failure, the nutrition prescription must respect the limitations of nonrenal organs (e.g., liver, lungs) and reconcile them with the CRRT-related adjustments.

### 5.3. Monitoring and Biomarkers of Adequacy

Any personalized approach must be guided by feedback. Reliance on static formulas without ongoing assessment risks both under- and overnutrition. Biomarkers and measurements that help guide adjustments include:

- Nitrogen balance/urea nitrogen appearance: by comparing nitrogen intake with excreted nitrogen (urine + CRRT effluent), one can estimate whether protein supply is sufficient.
- Plasma amino acid levels/profiles: these may reveal substrate depletion or disproportionate clearance.
- Metabolic markers: measures such as respiratory quotient (RQ) from indirect calorimetry, lactate, ketone levels, or substrate oxidation estimates help determine whether energy supply is anabolic or excessive.
- Micronutrient levels (e.g., B vitamins, trace elements): in patients on CRRT, deficiencies may evolve unless explicitly monitored. Wischmeyer et al. argue that in patients at risk (e.g., on RRT), micronutrient evaluation around ICU Day 5–7 should be considered [23].
- Clinical and laboratory parameters: trends in nitrogenous waste, acid–base status, glycemic control, electrolyte shifts, and signs of overfeeding (e.g., hypertriglyceridemia, elevated CO_2_ production) provide contextual feedback.

Moreover, some reviews argue the need for integrated biomarker panels rather than single metrics to gauge nutrition adequacy in critical care [24]. In CRRT patients, effluent sampling (i.e., measuring concentrations of amino acids, vitamins in the effluent) may offer direct insight into losses and thus guide compensation.

### 5.4. Strategies to Prevent Overfeeding and Underfeeding

Balancing between under- and overfeeding is delicate in CRRT patients, but several strategies can reduce the risk of extremes:

- Begin with a ramp-up or stepwise escalation: start at a fraction (e.g., 50–70%) of estimated energy and protein goals, then increase as tolerance is confirmed and metabolic stability ensues. This gradual approach helps prevent sudden metabolic overload. The trend in critical care nutrition supports progressive advances rather than full feeding from day 1 [25].
- Frequent reassessments and dynamic adjustment: daily (or more frequent) review of PN delivery vs. prescribed, biomarker trends, CRRT changes, and clinical course should guide up/down titration.
- Careful accounting of non-nutritional calories: subtract extra calories from citrate, dextrose in replacement solutions or propofol to avoid unintentional overfeeding—a frequent risk in ICU nutrition protocols [26].
- Safety thresholds and triggers: define maximum tolerable glucose infusion rates, lipid doses, total energy ceilings. If parameters (e.g., hyperglycemia, CO_2_ excess, hypertriglyceridemia) exceed thresholds, the feeding intensity must be reduced.
- Use modular supplementation: rather than remaking entire PN bags, clinicians can adjust individual modules (amino acids, electrolytes, vitamins) to fine-tune without systemic disruption.
- Avoid abrupt large changes: in response to a change in CRRT modality or dose, adjust PN gradually to allow metabolic adaptation rather than inducing shock.
- In patients with unstable multi-organ dysfunction, begin conservatively (slight underfeeding) but with aggressive protein provision, and escalate as the situation stabilizes.

By fusing CRRT-specific corrections, clinical phenotype adjustments, and tight monitoring, these strategies seek to deliver nutrition that matches the patient’s evolving requirements—not merely a static prescription.

## 6. Clinical Evidence and Outcomes

While the mechanistic rationale for personalized parenteral nutrition in CRRT is compelling, translating theory to improved clinical outcomes remains challenging. In this section we examine evidence regarding mortality, morbidity, ICU stay, muscle preservation, and the risks of metabolic complications or infections in critically ill patients receiving parenteral nutrition—especially in the context of renal replacement therapy [27].

### 6.1. Effects on Mortality, Morbidity, and ICU Stay

In general ICU populations (not specific to CRRT), randomized trials of total parenteral nutrition (TPN) versus standard care have shown no consistent mortality benefit. A classic meta-analysis combining 26 randomized trials (2211 patients) concluded that TPN did not significantly affect overall mortality in surgical or critically ill patients (RR ≈ 1.03, 95% CI 0.81–1.31) [3]. However, some benefit was seen in complication rates among malnourished subgroups, though effects were heterogeneous and influenced by study quality.

Critically ill patients with renal dysfunction represent a smaller and more complex subgroup, and evidence is even more sparse. Very few trials focus specifically on CRRT patients receiving PN. In mechanistic and observational series, nutrition adequacy (i.e., achieving closer to target energy/protein) is associated with shorter ICU length of stay, fewer days on mechanical ventilation, and reduced infectious complications, but the causality remains uncertain. For instance, Lopez-Delgado and colleagues observed that among ICU patients receiving PN, those with better protein delivery had shorter ICU duration and fewer days on ventilation compared to those with lower delivered protein, without a higher incidence of complications [28].

Meta-narrative reviews and nutritional guidelines suggest that in high-nutritional-risk patients (e.g., higher NUTRIC or SOFA scores), timely and adequate nutrition may improve outcomes more markedly than in low-risk patients [29]. In AKI patients undergoing CRRT, Lee et al. found that a higher prognostic nutritional index (PNI, combining albumin and lymphocyte count) was independently associated with better renal replacement therapy–free survival and lower 28- and 90-day mortality, even after adjusting for whether the patients received parenteral nutrition [30]. This suggests that baseline nutritional status (and by extension adequacy of nutritional therapy) is a meaningful prognostic marker in CRRT populations.

Taken together, while robust, prospective trials are lacking, existing observational and mechanistic evidence supports the concept that greater nutritional adequacy may translate into improved morbidity metrics (shorter ICU stay, ventilator-free days, fewer complications), and possibly modest mortality benefit in selected high-risk subsets.

### 6.2. Nutritional Adequacy and Muscle Preservation

One compelling rationale for aggressive nutritional strategies is preserving lean body mass and preventing ICU-acquired weakness or sarcopenia. In critical care settings, studies consistently show that cumulative protein deficits correlate with greater muscle loss (assessed by imaging or ultrasound), prolonged rehabilitation, and worse functional outcomes. Although data specific to CRRT patients are sparse, general ICU evidence strongly supports that higher protein delivery is associated with reduced muscle catabolism and improved nitrogen balance.

In CRRT, the continuous loss of amino acids amplifies the risk of muscle wasting. Some small observational cohorts measuring amino acid effluent losses and correlating them with nitrogen balance suggest that failure to account for these losses results in persistent negative nitrogen balance and muscle breakdown. For example, in a mechanistic investigation of CRRT metabolic aspects, Fishman and Singer characterized how unrecognized amino acid removal may negate the benefits of nominal protein dosing unless appropriately compensated [5]. Thus, achieving true nutritional adequacy requires not only delivering target protein but ensuring net positive nitrogen balance after extracorporeal losses.

In clinical practice, surrogate markers such as trends in serum urea nitrogen, nitrogen appearance, or periodic muscle thickness measurements (e.g., quadriceps ultrasound) can help infer effectiveness of protein regimens. In high-risk CRRT patients, combining higher protein targets with monitoring seems reasonable to support muscle preservation until further evidence becomes available.

In this context, the available clinical evidence strongly supports the need for and important role of supplemental parenteral nutrition (SPN) in CRRT patients. Observational studies demonstrate that enteral nutrition alone typically achieves only 60–70% of protein targets in this population, resulting in cumulative deficits of 5–7 g nitrogen/day despite aggressive feeding protocols. When comparing CRRT patients receiving enteral nutrition alone versus those receiving combined EN + PN, the latter group demonstrates improved nitrogen balance (−2 g/day vs. −5 g/day), better preservation of muscle mass measured by ultrasound, and reduced duration of mechanical ventilation. This evidence indicates that SPN serves a critical function in compensating for documented EN inadequacy and addressing the quantifiable extracorporeal nutrient losses unique to CRRT patients. While definitive large-scale RCTs are still needed, current data substantiate the importance of judicious SPN implementation when EN alone proves insufficient in this specialized population.

### 6.3. Metabolic Complications and Risk of Infections

Parenteral nutrition is not benign—especially in critically ill patients. The risks include hyperglycemia, refeeding syndrome, electrolyte derangements, overfeeding, liver dysfunction, and catheter-related bloodstream infections.

Overfeeding is a well-recognized hazard: New et al. [31] emphasized that excessive energy intake in CRRT patients can exacerbate CO_2_ production, hyperglycemia, lipogenesis, and metabolic derangements, potentially worsening outcomes. In classic critical care settings, overfeeding has been linked to higher mortality, longer ventilation times, and more complications. Thus the margin of safety is narrow, especially in CRRT where hidden caloric fluxes may already burden the system.

The clinical manifestations of overnutrition in CRRT patients deserve particular attention. Physiologically, excess caloric provision results in increased carbon dioxide production (typically >20–25 mL/kg/min), which may prolong ventilator dependence [31]. Biochemically, overfeeding manifests as hyperglycemia resistant to insulin therapy (blood glucose persistently >180–200 mg/dL despite escalating insulin requirements), hypertriglyceridemia (>300–400 mg/dL), and elevated liver function tests (ALT/AST elevation >2–3 times baseline) [31]. The biological consequences include increased oxidative stress through excess mitochondrial reactive oxygen species production, cellular injury from lipotoxicity, and immune dysfunction—particularly concerning in patients already metabolically vulnerable from critical illness and renal dysfunction. In the CRRT population specifically, these complications may be exacerbated by altered drug metabolism and impaired antioxidant clearance, potentially contributing to delayed recovery of renal function and prolonged CRRT dependence.

Infection risk, particularly catheter-associated bloodstream infection, is a major concern in PN administration. Parenteral nutrition requires intravascular access (usually central venous), and central line-associated sepsis is a known complication, with substantial attributable mortality. Early reports indicate infection rates of 0.5 to 2.0 per 1000 catheter-days, though modern sterile techniques reduce this risk. A narrative review cautions that metabolic and catheter-related complications remain key limiting factors in PN application in critically ill patients [32]. Some studies showed that early PN (compared to delayed) may increase infectious complications in trauma patients, emphasizing the need for caution [3].

Electrolyte shifts and refeeding syndrome represent additional risk, especially in malnourished patients. Refeeding-induced hypophosphatemia, hypokalemia, and hypomagnesemia can precipitate arrhythmias, muscle weakness, and neurologic dysfunction [31,32]. In CRRT patients, these shifts may be exaggerated given renal replacement of electrolytes, mandating vigilant monitoring and supplementation.

Finally, metabolic liver dysfunction (steatosis, cholestasis) can develop with prolonged PN, though in short-term ICU settings this is typically less relevant but still warrants awareness in long-duration PN use [3].

In sum, the clinician must balance the potential benefits of achieving nutritional adequacy (reduced muscle loss, better morbidity outcomes) against the risks of metabolic derangement and infection [28,32]. Meticulous catheter care, tight glucose control, gradual escalation of feeding, and frequent monitoring remain essential risk-mitigation strategies.

## 7. Practical Aspects and Implementation

Implementing personalized parenteral nutrition (PN) in critically ill patients undergoing CRRT requires not only physiological understanding but also practical strategies. These include decisions about when to initiate PN, how to integrate PN with enteral nutrition (EN), how to establish protocols and multidisciplinary collaboration, and how to address cost-effectiveness in the ICU.

### 7.1. Timing and Initiation of Parenteral Nutrition

The question of when to start PN in ICU patients is controversial. The ESPEN guidelines recommend that PN should be considered within 24–48 h if EN is contraindicated or insufficient, especially in malnourished patients [13]. Conversely, the EPaNIC trial, one of the largest randomized controlled trials in this field, found that delaying PN until day 8 reduced ICU infections, shortened mechanical ventilation, and decreased overall healthcare costs, without increasing mortality [33]. This has fueled debate between early versus late initiation.

It must be emphasized that when parenteral nutrition is clinically indicated, early prescription—ideally within the first 24–48 h of assessment—is crucial to prevent nutritional deterioration and support metabolic demands, particularly in high-risk patients. The decision to initiate PN should systematically consider several key factors: (1) the specific type of therapy being used (e.g., CRRT modality, effluent rate, and anticoagulation method), as each modality creates distinct nutrient clearance profiles; (2) the quantifiable and anticipated nutrient losses through the extracorporeal circuit; (3) the patient’s capacity for oral or enteral intake, including both volume tolerance and ability to meet calculated requirements; and (4) the patient’s mobilization status, as immobilization affects both metabolic needs and the risk of complications associated with nutritional support. Timely assessment of these factors facilitates appropriate early intervention while avoiding unnecessary PN in patients who may benefit from delayed initiation.

In CRRT patients, the balance is particularly delicate. Continuous nutrient losses and high catabolic stress suggest benefit from earlier PN, yet the risks of infection, overfeeding, and metabolic derangements argue for caution. Most experts recommend a hybrid approach: start early EN when feasible, assess adequacy daily, and initiate PN if by day 3 the patient is not achieving ≥60–70% of estimated needs. This individualized approach allows clinicians to balance substrate losses against the risks associated with PN [13,34].

### 7.2. Integration with Enteral Nutrition

EN is the preferred route whenever the gut is functional, due to its benefits for gut integrity, immune function, and lower infection risk [35]. In CRRT patients, however, EN alone is often insufficient, particularly under high catabolic loads. The strategy of supplemental PN (SPN)—initiating PN to cover the gap between delivered EN and calculated requirements—is recommended to optimize nutrition [36].

The integration must be dynamic. For example, as EN tolerance improves, PN should be tapered. In practice, EN can be started within 24–48 h, with PN added to reach targets by day 3–5 if EN alone cannot meet requirements [18]. This approach ensures that patients benefit from the physiological effects of EN while still achieving adequacy in protein and energy intake.

### 7.3. Protocols and Multidisciplinary Collaboration

Protocolized nutrition delivery and multidisciplinary teamwork improve outcomes. A study in a trauma ICU showed that involvement of a nutrition support team significantly improved caloric and protein delivery compared to routine care [37]. Similarly, implementation of structured PN protocols reduced errors, improved monitoring, and facilitated early identification of complications [38].

In CRRT patients, protocols should explicitly integrate dialysis prescription variables (e.g., effluent rate, modality, citrate use), nutritional goals, and monitoring thresholds (glucose, triglycerides, electrolytes). Daily rounds with intensivists, nephrologists, dietitians, pharmacists, and nursing staff help align CRRT settings with PN prescriptions and anticipate metabolic derangements.

### 7.4. Cost-Effectiveness Considerations

PN is resource-intensive, requiring specialized compounding, monitoring, and infection prevention strategies. The EPaNIC trial demonstrated that late initiation of PN reduced hospital costs by over €1000 per patient [33]. Other analyses confirm that avoiding unnecessary PN or reducing PN duration can lower expenditure without compromising outcomes [39].

At the same time, inadequately nourished patients incur costs from longer ICU stays, more infections, and prolonged CRRT dependence. Thus, the cost-effectiveness of PN in CRRT lies in personalized use: providing PN when EN is insufficient, tailoring doses to CRRT-specific losses, and discontinuing PN as soon as EN is tolerated. Future cost-effectiveness studies focused specifically on CRRT populations are needed, but current evidence supports a judicious, protocolized PN approach as both clinically and economically sound.

## 8. Future Directions

Looking ahead, three targeted innovations show particular promise for advancing parenteral nutrition in CRRT patients, though each requires rigorous validation before clinical implementation.

First, point-of-care analysis technologies could transform nutritional monitoring in CRRT. Miniaturized analyzers capable of measuring amino acids and micronutrients in CRRT effluent would enable precise quantification of extracorporeal losses [24]. These measurements, when paired with continuous metabolic monitoring (indirect calorimetry, glucose sensing), could create feedback systems where substrate infusion rates adjust to maintain target balances. Metabolomic profiles integrated with CRRT kinetic models would further refine nutrient matching beyond current static formulas [2].

Second, artificial intelligence and decision support systems address the complexity of managing nutrition during CRRT [40]. Kittrell et al. documented how AI applications, while currently limited to malnutrition screening and feeding tolerance assessment, increasingly demonstrate capacity to integrate multiple data streams for personalized nutritional prescriptions [24]. In CRRT specifically, decision support tools could provide real-time alerts when amino acid clearance exceeds PN delivery or when CRRT parameter changes necessitate formula recalibration. As outlined in a recent precision nutrition review, these systems can reduce human error and continuously monitor evolving needs while incorporating diverse clinical and laboratory inputs [7]. The key challenge remains developing transparent, interpretable models that earn clinician trust.

Third, targeted clinical trials must address specific knowledge gaps in CRRT nutrition. The current evidence base lacks randomized controlled trials specifically testing PN strategies in CRRT patients, with most guidance extrapolated from general critical care populations [7]. Future trials should stratify by CRRT modality and effluent dose while measuring clinically meaningful outcomes like mortality, ventilation duration, and renal recovery. While the ongoing “Supplemental Parenteral Nutrition in Critically Ill Adults” trial (NCT01847534) may offer insights applicable to CRRT patients [41], dedicated trials are needed to characterize micronutrient kinetics across different CRRT settings and validate biomarkers as predictors of nutritional status [8].

These focused innovations collectively support a transition from static PN dosing toward dynamic, feedback-driven nutritional therapy in CRRT—ultimately enabling personalized substrate support in this complex clinical setting.

## 9. Conclusions

CRRT induces substantial daily losses that must be compensated: 14–22 g of amino acids, water-soluble vitamins with sieving coefficients approaching 1.0, and trace elements including selenium and zinc. These losses necessitate increased protein targets of 1.8–2.5 g/kg/day—approximately 50% higher than standard ICU recommendations—to achieve nitrogen balance. Energy prescriptions require specific calibration to account for “hidden” calories. When using citrate anticoagulation (contributing 3–4 kcal/mmol) or glucose-containing dialysate, energy targets should be reduced by the calculated non-nutritional caloric load to prevent overfeeding complications. Timing of parenteral nutrition initiation should follow a defined protocol: early prescription (within 24–48 h) when indicated based on nutritional assessment, systematic evaluation of CRRT modality, documented losses, oral intake capacity, and mobilization status. A gradual escalation approach starting at 60–70% of targets minimizes metabolic complications. Also, implementation requires specific monitoring parameters: nitrogen balance measurements, effluent amino acid sampling when available, triglyceride levels at least twice weekly, and electrolyte monitoring every 4–8 h initially. Micronutrient levels, particularly selenium, zinc, and water-soluble vitamins, should be assessed weekly during prolonged therapy.

## Figures and Tables

**Table 1:** Key Nutritional Considerations and Adjustments for Patients on Continuous Renal Replacement Therapy.

Parameter	Standard ICU Target	CRRT-Adjusted Target	Rationale for Adjustment	Monitoring
Energy	20–25 kcal/kg/day	20–25 kcal/kg/day minus hidden calories	Account for citrate (3–4 kcal/mmol), propofol (1.1 kcal/mL), dialysate glucose	Indirect calorimetry, CO_2_ production, RQ
Protein/Amino Acids	1.2–1.5 g/kg/day	1.8–2.5 g/kg/day	Compensate for 14–22 g/day effluent losses	Nitrogen balance, effluent amino acid levels, urea appearance
Lipids	≤1.0 g/kg/day	≤1.5 g/kg/day (max 0.15 g/kg/h)	No significant CRRT clearance; use mixed emulsions	Triglycerides, liver function
Glucose	GIR ≤ 4 mg/kg/min	GIR ≤ 5 mg/kg/min adjusted for circuit flux	Variable losses/gains depending on dialysate	Blood glucose q1–2h initially
Water-soluble vitamins	Standard dose	1.5–2× standard dose	High clearance (SC ~1.0)	Serum levels if available
Trace elements	Standard dose	Selenium 100–200 μg/day, Zinc 10–15 mg/day	Significant effluent losses	Weekly levels
Electrolytes	Per serum levels	Increased supplementation	Continuous removal	Every 4–8 h initially

Abbreviations: GIR = glucose infusion rate; RQ = respiratory quotient; SC = sieving coefficient.

## Data Availability

No new data were created or analyzed in this study. Data sharing is not applicable to this article.

## Abbreviations

The following abbreviations are used in this manuscript:

PN	Parenteral nutrition
AKI	Acute kidney injury
CRRT	Continuous renal replacement therapy
CVVH	Continuous veno-venous hemofiltration
CVVHD	Continuous veno-venous hemodialysis
CVVHDF	Continuous veno-venous hemodiafiltration
EN	Enteral nutrition
ESPEN	European Society for Clinical Nutrition and Metabolism
GIR	Glucose infusion rate
ICU	Intensive care unit
MOF	Multi-organ failure
NUTRIC	Nutrition Risk in the Critically Ill
PNI	Prognostic nutritional index
SANRA	Scale for the Assessment of Narrative Review Articles
SPN	Supplemental parenteral nutrition
TPN	Total parenteral nutrition
